# De-escalation of axillary surgery and targeted axillary dissection following neoadjuvant chemotherapy: multicentre prospective regional audit

**DOI:** 10.1093/bjsopen/zraf172

**Published:** 2026-02-17

**Authors:** Mhairi Mactier, Laura Arthur, Louise Magill, Katherine Duncan, James Mansell, Esther Jennifer Campbell, Julie Doughty, Laszlo Romics

**Affiliations:** Wolfson Wohl Cancer Research Centre, College of Medicine and Veterinary Science, University of Glasgow, Glasgow, UK; General Surgery Department, Golden Jubilee National Hospital, Clydebank, UK; General Surgery Department, Royal Alexandra Hospital, Paisley, UK; General Surgery Department, University Hospital Wishaw, Glasgow, UK; General Surgery Department, Royal Alexandra Hospital, Paisley, UK; General Surgery Department, Gartnavel General Hospital, Glasgow, UK; General Surgery Department, Gartnavel General Hospital, Glasgow, UK; General Surgery Department, Gartnavel General Hospital, Glasgow, UK; General Surgery Department, Gartnavel General Hospital, Glasgow, UK

**Keywords:** breast neoplasms, axillary marker, targeted axillary surgery, neoadjuvant therapy

## Abstract

**Background:**

Emerging evidence supports axillary de-escalation in patients with clinically node-positive breast cancer with low-volume residual disease following neoadjuvant chemotherapy, avoiding axillary node clearance in selected patients. Targeted axillary dissection, which retrieves a known metastatic, clipped node alongside standard sentinel node biopsy aims to reduce false-negative rates. This study evaluated axillary surgery after neoadjuvant chemotherapy across NHS Greater Glasgow and Clyde, and examined 10-year trends.

**Methods:**

Patients with node-positive breast cancer receiving neoadjuvant chemotherapy between 2017 and 2024 were identified from multidisciplinary team records. Clinicopathological and surgical data were collected. Outcomes were compared using χ^2^ tests and logistic regression. Additional data from 2015–2016 were extracted from the Regional Cancer Registry.

**Results:**

Of 498 patients, primary axillary surgery included Magseed^®^-localized targeted axillary dissection (27.5%), wire-localized targeted axillary dissection (0.4%), non-localized targeted axillary dissection (7.0%), sentinel node biopsy (14.3%), and axillary node clearance (50.8%). The clipped node retrieval rate was 100% with Magseed^®^-localized and 91.4% with non-localized targeted axillary dissection; sentinel node concordance rates were 85.8 and 66.7%, respectively. Completion axillary node clearance was undertaken in 27 patients (11.0%) and was associated with an increased risk of complications including seroma, restricted shoulder movement, and wound infection, compared with de-escalated surgery (odds ratio (OR) 2.88, 95% confidence interval (CI) 1.28 to 6.49; *P* = 0.011) and upfront axillary node clearance (OR 1.86, 95% CI 1.27 to 2.72; *P* = 0.001). Use of axillary de-escalation increased over 10 years, surpassing 50% recently (χ²(4) = 25.3, *P* < 0.001).

**Conclusion:**

Targeted axillary dissection enables safe de-escalation of axillary surgery in patients with low-volume residual disease. Localization enhances clipped node retrieval. Completion axillary node clearance carries higher morbidity, reinforcing the need for careful patient selection.

## Teaser

This study adds to growing evidence supporting axillary de-escalation in patients with node-positive breast cancer who response well to neoadjuvant chemotherapy. The present findings highlight the role of localization techniques in improving clipped node retrieval and reducing false-negative rates. The study also examined the indications and complications associated with completion axillary node clearance, highlighting the need for clearer criteria for upfront clearance and ‘getting it right first time’.

## Introduction

Following neoadjuvant chemotherapy (NACT), up to 70% patients with node-positive breast cancer have a pathological complete response (pCR) in the axilla^1,2^. Despite this, 60% of units across the UK offer axillary node clearance (ANC) for patients with a positive axilla at diagnosis, regardless of response to NACT^3^. Furthermore, the current National Institute for Health and Care Excellence guidelines^4^ do not address changes in surgical management following neoadjuvant treatment. ANC carries substantially higher morbidity than less extensive approaches, with lymphoedema rates of 20–25% compared with 5–10% after sentinel node biopsy (SNB) and near zero in early targeted axillary dissection (TAD) series, alongside higher rates of shoulder stiffness, sensory loss, and seroma^5–8^. The importance of addressing alternative treatment modalities was highlighted as the number one priority by patients and clinicians in the 2021 James Lind Alliance for breast surgery^9^.

Emerging evidence has demonstrated a shift towards de-escalation of axillary surgery in select patients after NACT, avoiding the need for ANC^10,11^. SNB has been shown to achieve unacceptable false-negative rates (FNRs)^12^. TAD, initially described by Caudle *et al.*^13^, comprises a combination of targeted lymph node biopsy to retrieve a known metastatic clipped node alongside standard SNB. TAD has an acceptable FNR, and additional localization techniques targeting the clipped node can reduce the FNR further, with reported rates of as low as 2%^13,14^.

There is, however, a lack of real-world data, as most published evidence so far is based on single-centre reviews. Hence, a regional audit in Scotland’s largest Health Board was carried out. Across NHS Greater Glasgow and Clyde, TAD was introduced for patients with node-positive disease who have evidence of clinical and/or radiological response in the axilla following NACT. This study reports on experience of Magseed^®^(Endomagnetics, Cambridge, UK)-localized TAD, compares outcomes of de-escalated axillary surgery with those of ANC, explores the diagnostic value and complication risk associated with completion ANC (cANC), and reviews trends in surgical management of the axilla following NACT over the past 10 years.

## Methods

### Patient selection

All patients with node-positive invasive breast cancer who underwent NACT between 1 January 2017 and 31 December 2024 were identified from prospectively maintained multidisciplinary team (MDT) records across all breast units in NHS Greater Glasgow and Clyde Health Board (Gartnavel General Hospital and Stobhill Hospital, Glasgow, and Royal Alexandra Hospital, Paisley). All patients with suspicious nodes on clinical examination and/or imaging underwent core biopsy to confirm the presence of nodal metastases. Suspicious or abnormal axillary nodes were defined as those with a cortex > 3 mm or loss of normal morphology. Breast mammography, ultrasonography, and magnetic resonance imaging (MRI) were carried out at baseline before NACT. Response to treatment was monitored in accordance with departmental protocol. Post-NACT imaging was reviewed at a MDT meeting before surgery. Patients considered to have a complete or excellent radiological response to NACT were offered de-escalated axillary surgery, whereas those with a poor response were offered ANC; cANC was considered in most patients with residual metastatic lymphadenopathy after de-escalated axillary surgery, with decision-making similar to that for patients with a positive SNB. To review trends in clinical practice over a 10-year period, additional data were extracted from the West of Scotland Cancer Registry for all patients with node-positive disease who received NACT from 1 January 2015 to 31 December 2016.

### Surgical technique

For patients suitable for and agreeable to de-escalated axillary surgery, a variety of techniques were used over the years. In the early stages, four-node sampling with dual-technique SNB was performed. Marking the abnormal node with a clip at the time of initial biopsy varied between units (2017–2021); where more than one positive node was present, the marking clip was placed in the largest metastatic node. TAD was then carried out, retrieving the known metastatic clipped node alongside a standard SNB, and the technique was supplemented with an intraoperative specimen X-ray to confirm clip retrieval. For the purpose of the present study, this technique was termed ‘non-localized TAD’. Between September and December 2017, one unit trialled wire-localized TAD. Thereafter, between October and December 2020, Magseed^®^-localization for involved nodes was adopted in two of three units, and has since been adopted by the third unit in the present study. Following completion of NACT, the clipped node was localized under ultrasound guidance with Magseed^®^. On the day of surgery, Magseed^®^-guided dissection of the axilla was performed to retrieve the localized node using a Sentimag^®^ probe (Endomagnetics), and again an intraoperative specimen X-ray was obtained to confirm Magseed^®^ and clip retrieval. Further sentinel nodes were retrieved, with the aim of removing a minimum of three nodes altogether. Standard techniques were used for patients undergoing ANC. Histopathological analysis confirmed evidence of nodal metastases; in this study, macrometastases only (> 2 mm) were considered node-positive. Evidence of isolated tumour cells or micrometastases was considered to indicate node-negative disease.

### Clinical data analysis

Data on patient demographics, tumour characteristics, baseline and response to treatment imaging, neoadjuvant treatment regimens, surgical details, and postoperative complications were collected from electronic patient records. Imaging characteristics were grouped to mirror clinical decision-making (normal; 1 abnormal node; ≥ 2 abnormal nodes). Complications were assessed by severity based on the Clavien–Dindo classification^15^, and by location. Minor complications (Clavien–Dindo grades I–II) included conservatively managed localized wound complications (surgical site infection, prolonged wound healing, seroma), restricted shoulder range of movement, and/or pain issues. Major complications (Clavien–Dindo grade ≥ III) included advanced surgical site infection requiring intravenous antibiotics and/or washout, haematoma requiring surgical evacuation and/or re-exploration of immediate breast reconstruction. Breast complications included those localized to breast excision wounds and/or reconstruction complications, whereas axillary complications included axillary wound infections/seroma, restricted shoulder movement, arm paraesthesia, and lymphoedema. Systemic complications included non-wound-related infection, venous thromboembolism, and/or delirium. Patient-reported lymphoedema at limited follow-up was also assessed separately. Survival data were obtained from the West of Scotland Cancer Registry for all patients, including date and cause of death.

### Ethical considerations

NHS Greater Glasgow and Clyde Caldicott approval was obtained for access to electronic patient records. The Regional Information Government Framework at West of Scotland Cancer Network safeguards the sharing of data for clinical audit purposes^16^. All analysis was undertaken using pseudonymized data. Principles of Good Clinical Practice and the Declaration of Helsinki were adhered to.

### Statistical analysis

Patient demographics, clinicopathological variables, and axillary surgical techniques are presented as categorical variables. Relationships between variables were examined using the χ^2^ test and logistic regression analysis. All tests were two-sided, and *P* < 0.050 was considered statistically significant. Statistical analysis was carried out using SPSS^®^ version 29.1 (IBM, Armonk, NY, USA).

## Results

Some 498 patients with node-positive invasive breast cancer who received NACT between 1 January 2017 and 31 December 2024 were identified. Of these, 137 patients underwent Magseed^®^-localized TAD (27.5%), 2 underwent wire-localized TAD (0.4%), 35 had non-localized TAD (7.0%), 71 underwent SNB (14.3%), and 253 had ANC (50.8%). Age at diagnosis (*P* = 0.803), mode of presentation (*P* = 0.224), clinical tumour size (cT; *P* = 0.557), clinical nodal presentation (cN; *P* = 0.290), histological subtype (*P* = 0.626), and grade (*P* = 0.695) were evenly distributed between surgical techniques (*Table 1*). However, tumour biology was associated with primary axillary surgery (*P* = 0.002). Patients with human epidermal growth factor receptor 2 (HER2)-positive tumours were more likely to undergo de-escalated procedures, reflecting treatment responsiveness. The overall survival (OS) rate was 84.4%, and the breast cancer-specific survival (BCSS) rate was 85.6%, with median follow-up of 4.75 (range 1.03–8.48) years.

**Table 1:** Patient and tumour characteristics according to primary axillary surgery technique

	Axillary technique					P*
	Localized TAD (*n* = 139)	Non-localized TAD (*n* = 35)	SNB (*n* = 71)	ANC (*n* = 253)	Total (*n* = 498)	
**Age at diagnosis (years)**						0.803
< 50	51 (36.7%)	10 (28.6%)	22 (31.0%)	97 (38.3%)	180 (36.1%)	
50–69	75 (54.0%)	21 (60.0%)	44 (62.0%)	136 (53.8%)	276 (55.4%)	
≥ 70	13 (9.4%)	4 (11.4%)	5 (7.0%)	20 (7.9%)	42 (8.4%)	
Median (range)	53 (25–79)	57 (33–74)	54 (28–76)	53 (24–79)	53 (24–79)	
**Mode of presentation**						0.224
Screening	15 (10.8%)	6 (17.1%)	10 (14.1%)	19 (7.5%)	50 (10.0%)	
Symptomatic	121 (87.1%)	27 (77.1%)	60 (84.5%)	230 (90.9%)	438 (88.0%)	
Other	3 (2.2%)	2 (5.7%)	1 (1.4%)	4 (1.6%)	10 (2.0%)	
**cT category**						0.557
cT1	24 (17.3%)	6 (17.1%)	14 (19.7%)	32 (12.6%)	76 (15.3%)	
cT2	61 (43.9%)	15 (42.9%)	20 (42.3%)	98 (38.7%)	204 (41.0%)	
cT3	40 (28.8%)	10 (28.6%)	17 (23.9%)	79 (31.2%)	146 (29.3%)	
cT4	14 (10.1%)	4 (11.4%)	10 (14.1%)	44 (17.4%)	72 (14.5%)	
**cN category**						0.290
cN1	123 (88.5%)	32 (91.4%)	67 (94.4%)	215 (85.0%)	437 (87.8%)	
cN2	9 (6.5%)	3 (8.6%)	2 (2.8%)	19 (7.5%)	33 (6.6%)	
cN3	7 (5.0%)	0 (0%)	2 (2.8%)	19 (7.5%)	28 (5.6%)	
**Tumour subtype**						0.626
Ductal	129 (92.8%)	32 (91.4%)	63 (88.7%)	222 (87.7%)	446 (89.6%)	
Lobular	7 (5.0%)	3 (8.6%)	4 (5.6%)	14 (5.5%)	28 (5.6%)	
Mixed/other	1 (0.7%)	0 (0%)	2 (2.8%)	10 (4.0%)	13 (2.6%)	
Unknown	2 (1.4%)	0 (0%)	2 (2.8%)	7 (2.8%)	11 (2.2%)	
**Tumour grade**						0.695
1	3 (2.2%)	0 (0%)	0 (0%)	5 (2.0%)	8 (1.6%)	
2	50 (36.0%)	12 (34.2%)	26 (36.6%)	78 (31.7%)	166 (33.3%)	
3	81 (58.3%)	23 (65.7%)	42 (59.2%)	163 (66.3%)	309 (62.0%)	
Unknown	5 (3.6%)	0 (0%)	3 (4.2%)	6 (2.4%)	15 (3.0%)	
**Tumour biology**						0.002
ER+ HER2–	30 (21.6%)	6 (17.1%)	27 (38.0%)	79 (31.2%)	142 (28.5%)	
ER+ HER2+	49 (35.3%)	8 (22.9%)	17 (23.9%)	65 (25.7%)	139 (27.9%)	
TNBC	31 (22.3%)	10 (28.6%)	15 (21.1%)	81 (32.0%)	137 (27.5%)	
ER– HER2+	29 (20.9%)	11 (31.4%)	12 (16.9%)	28 (11.1%)	80 (16.1%)	

Values are *n* (%) unless otherwise stated. TAD, targeted axillary dissection; SNB, sentinel node biopsy; ANC, axillary node clearance; cT/cN, clinical stage at diagnosis/before treatment; ER, oestrogen receptor; HER2, human epidermal growth factor receptor 2; TNBC, triple-negative breast cancer; *χ^2^ test.

### Localized TAD

Altogether 148 patients had a biopsied axillary node marked with a clip. Of these, 139 underwent localization, as the clipped node was not seen on axillary ultrasonography in 9 patients (6.1%) after NACT. Among patients who had localization, 137 underwent Magseed^®^-localized TAD. The clipped node was identified and retrieved in all patients. The Magseed^®^ was identified within the clipped node in 125 patients (91.2%); the seed was found in adjacent axillary tissue in 10 patients (7.3%), displaced high up in the axilla in 1 (0.7%), and was not identified in 1 patient (0.7%). Dual-mapping technique with blue-dye and radioisotope was used in 127 patients (92.7%). The rate of clipped node/sentinel node concordance was 85.8% (109 of 127). The median nodal harvest was 4 (range 1–11). A pCR was demonstrated in 70 patients (51.1%), whereas 8 (5.8%) had non-clipped nodal involvement. Thirteen patients (9.4%) proceeded to cANC, with additional nodal involvement identified in six (4.4%). Two patients underwent wire-localized TAD. The clipped node was identified and retrieved in both patients. Clipped node/sentinel node concordance was identified in neither; both had non-clipped nodal involvement so went on to have cANC. No additional positive lymphadenopathy was identified at cANC. Among patients who underwent localized TAD, both OS and BCSS rates were 97.3%.

### Non-localized TAD

A total of 35 patients underwent non-localized TAD. The clipped node was retrieved in 32 patients (91.4%). A dual-mapping technique was used in 33 patients (94.3%), with a clipped node/sentinel node concordance rate of 66.7% (22 of 33). The median nodal harvest was 4 (range 2–12). Twenty-one patients (60.0%) achieved a pCR, whereas two (5.7%) had non-clipped nodal involvement. Eight patients (22.9%) subsequently underwent cANC, with additional nodal involvement identified in five (14.3%). Among patients who underwent non-localized TAD, both OS and BCSS rates were 86.0%.

### SNB

A total of 71 patients underwent SNB, with a dual-mapping technique used in 58 (81.7%). The median nodal harvest was 4 (range 1–10). Thirty-nine patients (54.9%) had a pCR. Four proceeded to cANC (5.6%), with additional nodal involvement identified in all of these patients. Among patients who underwent SNB, the OS and BCSS rates were 82.7% and 84.0%, respectively.

### ANC

A total of 253 patients underwent ANC. The median nodal harvest was 12 (range 3–33), whereas median nodal involvement was only 2 (range 0–29). Seventy patients (27.7%) had a pCR in the axilla after NACT. Among patients who underwent upfront ANC, OS and BCSS rates were 74.6% and 76.7%, respectively.

### De-escalated *versus* clearance surgery

Axillary surgery was de-escalated in 245 patients, 27 of whom subsequently had cANC. Thus, 218 patients (43.8%) underwent TAD/SNB with or without localization techniques, termed ‘de-escalated axillary surgery’, and 280 (56.2%) underwent ANC/cANC. Screen-detected disease was more common among those who had de-escalated axillary surgery (*P* = 0.026), whereas the two groups had similar age ranges (*P* = 0.531), cT (*P* = 0.138) and cN (*P* = 0.096) categories, histological subtypes (*P* = 0.447), and grade (*P* = 0.895) (*Table 2*). Tumour biology was associated with final surgery (*P* < 0.001); overall, patients with HER2-positive tumours were more likely to have de-escalation of axillary surgery, whereas those with oestrogen receptor (ER)-positive HER2-negative tumours, and triple-negative breast cancer (TNBC) were more likely to undergo clearance surgery. In addition, both breast and axillary pCR were more common in the de-escalation group compared with the ANC/cANC group (ypT0: 50.9% *versus* 25.9%, *P* < 0.001; ypN0: 59.6% *versus* 25.0%, *P* < 0.001).

**Table 2:** Patient and tumour characteristics according to final axillary surgery

	Axillary technique		P*
	De-escalation surgery (*n* = 218)	ANC/cANC (*n* = 280)	
**Age at diagnosis (years)**			0.531
< 50	73 (33.5%)	107 (38.2%)	
50–69	125 (57.3%)	151 (53.9%)	
≥ 70	20 (9.2%)	2 (7.9%)	
Median (range)	54 (25–79)	53 (24–79)	
**Mode of presentation**			0.026
Screen-detected	30 (13.8%)	20 (7.1%)	
Symptomatic	182 (83.5%)	256 (91.4%)	
Other	6 (2.8%)	4 (1.4%)	
**cT category**			0.138
cT1	40 (18.3%)	36 (12.9%)	
cT2	93 (42.7%)	111 (39.6%)	
cT3	60 (27.5%)	86 (30.7%)	
cT4	25 (11.5%)	47 (16.8%)	
**cN category**			0.096
cN1	198 (90.8%)	239 (85.4%)	
cN2	13 (6.0%)	20 (7.1%)	
cN3	7 (3.2%)	21 (7.5%)	
**Tumour subtype**			0.447
Ductal	198 (90.8%)	248 (88.6%)	
Lobular	13 (6.0%)	15 (5.4%)	
Other	3 (1.4%)	10 (3.6%)	
Unknown	4 (1.8%)	7 (2.5%)	
**Tumour grade**			0.895
1	3 (1.4%)	5 (1.8%)	
2	74 (33.9%)	92 (32.9%)	
3	133 (61.0%)	176 (62.9%)	
Unknown	8 (3.7%)	7 (2.5%)	
**Tumour biology**			< 0.001
ER+ HER2−	52 (23.9%)	90 (32.1%)	
ER+ HER2+	69 (31.7%)	70 (25.0%)	
TNBC	47 (21.6%)	90 (32.1%)	
ER–HER2+	50 (22.9%)	30 (10.7%)	
**ypT category**			< 0.001
pCR	109 (50.9%)	72 (25.9%)	
ypT1	51 (23.8%)	63 (22.7%)	
ypT2	39 (18.2%)	86 (30.9%)	
ypT3	15 (7.0%)	57 (20.5%)	
**ypN category**			< 0.001
pCR	130 (59.6%)	70 (25.0%)	
ypN1	83 (38.1%)	103 (36.8%)	
ypN2	5 (2.3%)	65 (23.2%)	
ypN3	0 (0%)	42 (15.0%)	

Values are *n* (%) unless otherwise stated. cANC, completion axillary node clearance; cT/cN, clinical stage before neoadjuvant chemotherapy; ER, oestrogen receptor; HER2, human epidermal growth factor receptor 2; TNBC, triple-negative breast cancer; pCR, pathological complete response; ypT/ypN, pathological stage after therapy. *χ^2^ test.

Baseline and treatment-response nodal characteristics are summarized in *Table 3*. Rates of clinically palpable nodes and nodal involvement on baseline imaging were comparable between the groups. Response to NACT was monitored in accordance with departmental policy, including investigations carried out mid-treatment (MRI only, 97 patients) and/or at the end of treatment (ultrasonography, 285; MRI, 288). Regardless of modality, normal imaging after NACT was more common in the de-escalation group than the cANC and ANC groups, mirroring histopathological findings described previously (ultrasonography: 84.8% *versus* 52.2% *versus* 18.3%, respectively, *P* < 0.001; MRI: 58.2% *versus* 47.6% *versus* 31.0%, respectively, *P* < 0.001).

**Table 3:** Clinical and radiological characteristics for patients who underwent de-escalated axillary surgery, ANC, and cANC

	Axillary technique			P*
	De-escalation surgery (*n* = 218)	ANC (*n* = 253)	cANC (*n* = 27)	
Clinically palpable nodes at baseline	122 (56.0%)	163 (64.4%)	14 (51.9%)	0.117
**Baseline ultrasonography**				0.204
Normal	4 (1.9%)	6 (2.4%)	1 (3.7%)	
1 node	80 (37.0%)	68 (26.9%)	9 (33.3%)	
≥ 2 nodes	132 (61.1%)	179 (70.8%)	17 (63.0%)	
**Baseline MRI**				0.068
Normal	25 (13.2%)	14 (6.7%)	3 (15.0%)	
1 node	54 (28.4%)	46 (22.1%)	5 (25.0%)	
≥ 2 nodes	111 (58.4%)	148 (71.2%)	12 (60.0%)	
**Response to treatment, ultrasonographic assessment**				< 0.001
Normal	162 (84.8%)	13 (18.3%)	12 (52.2%)	
1 node	21 (11.0%)	29 (40.8%)	5 (21.7%)	
≥ 2 nodes	8 (4.2%)	29 (40.8%)	6 (26.1%)	
**Response to treatment, MRI assessment**				< 0.001
Normal	103 (58.2%)	58 (31.0%)	10 (47.6%)	
1 node	28 (15.8%)	48 (25.7%)	5 (23.8%)	
≥ 2 nodes	46 (26.0%)	81 (43.3%)	6 (28.6%)	

Values are *n* (%) unless otherwise stated. cANC, completion axillary node clearance; MRI, magnetic resonance imaging. *χ^2^ test.

Following cANC, 15 of 27 patients had additional nodal involvement; therefore, 15 of 218 (6.9%) were inadequately staged following de-escalated axillary surgery after NACT. Although over half of patients (15 of 27) had additional nodal involvement on cANC, the median additional nodal yield was only 1 (range 0–9). This varied over the study timeframe, and no trend was identified. In addition, it was not possible to identify any clinicopathological factors that increased the likelihood of cANC.

The total number of complications was higher after cANC than de-escalation surgery or ANC (*P* = 0.001) (*Table 4*). Patients had a nearly three-fold higher risk of developing a complication after cANC compared with de-escalation surgery (odds ratio (OR) 2.88, 95% confidence interval (CI) 1.28 to 6.49; *P* = 0.011). The complication risk after cANC was higher than that after primary ANC compared with de-escalation surgery (OR 1.86, 95% CI 1.27 to 2.72; *P* = 0.001) (*Table 5*). Further analysis of complications by severity and location revealed that minor complications and axillary complications were independently associated with cANC compared with de-escalation surgery or ANC (minor complications: 48.1% *versus* 23.4% *versus* 28.5%, respectively, *P* = 0.021; axillary complications: 48.1% *versus* 15.1% *versus* 28.5%, respectively, *P* < 0.001).

**Table 4:** Complications following de-escalation surgery, ANC, and cANC

	Axillary technique			P*
	De-escalation surgery (*n* = 218)	ANC (*n* = 253)	cANC (*n* = 27)	
**Complication by severity**				
Minor	51 (23.4%)	72 (28.5%)	13 (48.1%)	0.021
Major	6 (2.8%)	27 (10.7%)	1 (3.7%)	0.002
**Complication by site**				
Breast	25 (11.5%)	37 (14.6%)	1 (3.7%)	0.21
Axilla	33 (15.1%)	72 (28.5%)	13 (48.1%)	< 0.001
Systemic	0 (0%)	2 (0.8%)	1 (3.7%)	0.055
Lymphoedema	6 (2.8%)	21 (8.3%)	1 (3.7%)	0.030
Total complications	66 (30.3%)	113 (44.7%)	15 (55.6%)	0.001

Values are *n* (%) unless otherwise stated. cANC, completion axillary node clearance. *χ^2^ test.

**Table 5:** Logistic regression analysis comparing complications following de-escalation surgery, ANC, and cANC

	Odds ratio (95% confidence interval)	*P value*
De-escalation surgery	1.00 [reference]	
ANC	1.86 (1.27 - 2.72)	0.001
cANC	2.88 (1.28 - 6.49)	0.011

Values in parentheses are 95% confidence intervals. cANC, clearance axillary node clearance.

### Trends over 10 years

**Fig. 1:**
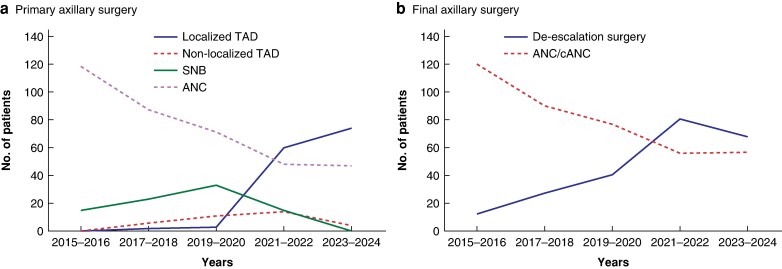
Changes over time in primary and final axillary surgery for node-positive disease after neoadjuvant chemotherapy **a** Primary and **b** final axillary surgery. TAD, targeted axillary dissection; SNB, sentinel node biopsy; ANC, axillary node clearance; cANC, completion axillary node clearance

In 2015–2016, before the introduction of TAD, most patients underwent ANC (118, 88.7%) and few had SNB (15, 11.3%). Two patients had cANC; therefore, 120 patients (90.2%) underwent clearance surgery and axillary surgery was de-escalated in 13 (9.8%) after NACT. By comparison, in the latter 2 years (2023–2024), axillary surgery was de-escalated in 68 patients (54.4%), with less than 50% undergoing clearance surgery. The rate of final axillary surgery differed significantly across the time periods (χ²(4) = 25.3, *P* < 0.001), reflecting a clear trend towards increasing use of de-escalation over time. Primary and final surgery trends are shown in *Fig. 1*.

## Discussion

This regional audit has provided real-world data supporting de-escalation surgery of the axilla in patients with node-positive breast cancer who have a good response to NACT. To the authors’ knowledge, this is the largest case series for Magseed^®^-localized TAD after NACT, providing prospective, multisite data that address a gap in the current literature^17^. In addition, this study has provided further insight into clinicopathological features associated with clearance surgery, as well as the increased risk of complications associated with cANC compared with primary ANC, emphasizing the importance of appropriate patient selection and ‘getting it right first time’.

Magseed^®^-localized TAD provided a higher clipped node retrieval rate than non-localized TAD (100% *versus* 91.4%), and the rate of clipped node/sentinel node concordance was also improved with localization (85.8% *versus* 66.7%). There was no difference in median nodal harvest; however, the cANC rate following Magseed^®^-localized TAD was lower than that after non-localized TAD (9.4% *versus* 22.9%), as was additional nodal involvement (4.4% *versus* 14.3%). These findings demonstrate improved accuracy of axillary staging using localization techniques, in agreement with the existing literature^13,14,18^.

The timing of localization has also been reviewed in recent literature^19^. In the present study, the Magseed^®^ was placed after NACT (2-stage approach). There were an additional 9 patients in whom the clipped node could not be visualized and so Magseed^®^-localization was not feasible in 9 of 148 (6.1%). This decreased over time suggestive of an introductory learning curve (12.8% in 2022 *versus* 4.4% in 2024). The SenTa study^20^ found that localization after NACT was more successful in patients with visible residual disease. The MDT approach in the present study was to select patients with minimal or low-volume residual disease for TAD, which may also explain the rate of failed localization. In addition, the ULTRACOR TWIRL® breast tissue marker (Becton, Dickinson and Company, Georgia, USA) Clip, which was found to demonstrate best ultrasound visibility in the SMART trial^21^, has now been adopted for routine use in this patient group. Furthermore, Magseed^®^ displacement from the clipped node has also reduced over time, mirroring the learning curve for this technique (9.5% in 2021 *versus* 4.7% in 2024). Although Magseed^®^ displacement did not affect clipped node retrieval in this study, it did highlight to the team the importance of considering failed node localization when consenting patients.

Comparing clinicopathological features by final surgery, it is not surprising that screen-detected tumours were more common in the de-escalation group (*P* = 0.026); however, most patients (88%) in this study were symptomatic at presentation. Screen-detected tumours are associated with better prognosis^22,23^, suggesting appropriate de-escalation of axillary surgery in the patient cohort. In addition, HER2-positive tumours were consistently associated with a pCR in the breast and axilla, and de-escalation surgery was more likely in these patients. There were lower rates of pCR among patients with TNBC in the study cohort, which explains the trend towards clearance surgery in this patient group.

When analysing radiological response to NACT, it is apparent that patients who underwent cANC had intermediate characteristics between those who had de-escalation surgery and those who had primary ANC. Further analysis revealed that abnormal nodes on ultrasonography were more common among patients who had cANC compared with de-escalation surgery (47.8% *versus* 15.2%; *P* < 0.001), but not when abnormal nodes were compared on MRI (52.4% *versus* 41.8%; *P* = 0.567). In the present study, MRI had higher sensitivity than ultrasonography (68.6% *versus* 45.9%) indicating that the decision for cANC was based on pathological response instead of radiological findings. By comparison, specificity was higher for ultrasonography compared with MRI (79.7% *versus* 63.5%). Some 31.0% of patients who underwent upfront ANC had normal MRI findings after NACT, highlighting the challenges faced by clinicians when selecting patients suitable for de-escalated axillary surgery, and the need for robust evidence and subsequent clinical guidance. Current imaging modalities are limited in accuracy of assessment of the axillary response to NACT and this should be addressed in future research^10,24,25^. Some small studies^26,27^ have suggested that contrast-enhanced spectral mammography may serve as an adjunct or alternative to MRI, providing comparable sensitivity for detecting residual disease while potentially improving patient tolerability and accessibility; however, larger studies are needed to confirm these findings.

In the present study, over 50% of patients who underwent cANC had additional nodal involvement, though the median additional nodal yield was only 1. Although further surgery is justified, understanding of the oncological benefit is unclear and all 27 patients went on to have nodal radiotherapy. Early follow-up studies reported comparable survival rates (disease-free survival (DFS): hazard ratio (HR) 1.26, 95% CI 0.27 to 5.87, *P* = 0.77; OS: HR 0.81, 95% CI 0.15 to 3.83)^28^ with low rates of axillary recurrence (1.0% (95% CI 0.49 to 2.0))^29^ following omission of ANC for patients with ycN0 disease after NACT in the presence of nodal radiotherapy. Recent results from the NSABP-51 Radiation Therapy Oncology Group 1304 trial^30^ have demonstrated no additional oncological benefit from nodal radiotherapy in patients with ypN0 disease after NACT, albeit just under 50% of patients in this study underwent ANC. Omission of ANC and nodal radiotherapy has been studied less; the ongoing UK ATNEC trial^31^ is randomizing patients with ycN0 disease to standard treatment (ANC or nodal radiotherapy) or no further axillary treatment and will provide level I evidence to support management of patients with cN+ tumours converted to ycN0 after NACT. Meanwhile, the management of ongoing nodal involvement after NACT is less clear. Retrospective data from the NSABP B40 and B41 trials^32^ have demonstrated no difference in 5-year DFS or OS for patients with ypN+ tumours between groups undergoing SNB only, SNB + ANC, and ANC only after NACT, with most of these patients having nodal radiotherapy. Meanwhile, the ongoing Alliance A011202 trial^33^ is randomizing patients with ypN+ disease to ANC or level I–II axillary radiotherapy, with all patients having regional nodal radiation to level III nodes and the supraclavicular fossa, to evaluate whether omission of ANC is non-inferior to radiotherapy in treatment of ypN+ disease after NACT. Finally, tailored axillary surgery (TAS) is an extension of TAD that involves selective removal of positive lymph nodes only. The TAXIS trial^34^ is randomizing patients to TAS followed by ANC and nodal radiotherapy excluding the axilla, or TAS with full axillary nodal radiotherapy. The hypothesis of the study is that TAS and full axillary nodal radiotherapy is non-inferior to ANC in terms of DFS in patients with clinically node-positive disease receiving neoadjuvant systemic therapy. This trial may offer an alternative to ANC for patients with a higher burden of residual axillary disease after NACT.

The present study has demonstrated a movement towards de-escalation of axillary surgery in patients with a good response to NACT over the past 10 years. Avoidance of ANC has been shown in over 50% patients in the latter years, with a significant impact in reducing patient morbidity, addressing the number one priority for patients in the management of breast cancer^13^. These findings support previous evidence that localization techniques improve the clipped-node retrieval rate during TAD, as well as the limitations of current imaging modalities in selecting patients suitable for de-escalation of axillary surgery. In the last 12 months, there was a marginal increase in the number of cANCs performed. It is thought that this is reflective of extending TAD to patients with more advanced nodal disease after NACT in an effort to avoid upfront ANC. However, this study demonstrated an increased risk of complications following cANC compared with upfront ANC, highlighting a need for better clinical guidelines for assessing response to NACT and suitable patient selection.

Limitations of this study include its retrospective nature, and lack of oncological follow-up data. There was a lack of consistency in the reporting of radiological findings after NACT which made interpretation of clinical response in association with post-NACT pathology challenging. Lastly, knowledge and insight into patient decision-making regarding different surgical options is lacking. Patient-reported outcomes were not assessed in this study; however, ongoing studies including ATNEC^31^ and Alliance A011202^33^ are addressing this important aspect of axillary management.

This study has demonstrated safe use of localized TAD, with a clipped-node retrieval rate of 100%. Use of de-escalation axillary surgical techniques enabled avoidance of ANC in over 50% of patients in the later years. In addition, nearly 30% of patients who underwent ANC had a pCR in the axilla, suggesting scope for further de-escalation of axillary surgery. The increased risk of complications associated with cANC has emphasizing the need to ‘get it right first time’. Further research to improve the assessment of response to NACT and appropriate patient selection for de-escalation of axillary surgery is required.

## Data Availability

The authors are willing to make their data, analytical methods, and study materials available to other researchers upon reasonable request, including relevant ethical and legal permissions to the corresponding author.
